# Doping Effects on Multivalence States, Electronic Structure, and Optical Band Gap in LaCrO_3_ under Varied Atmospheres: An Integrated Experimental and Density Functional Theory Study

**DOI:** 10.1021/acsaelm.4c02359

**Published:** 2025-03-07

**Authors:** Edward M. Sabolsky, Javier A. Mena, Víctor Mendoza-Estrada, Rafael González-Hernández, Katarzyna Sabolsky, Konstantinos Sierros

**Affiliations:** † Department of Mechanical, Materials, and Aerospace Engineering, West Virginia University, Morgantown, West Virginia 26506, United States; ‡ Grupo de Investigación en Física Aplicada, Departamento de Física, Universidad del Norte, Barranquilla 081001, Colombia; § Facultad de Ciencias, Educación, Artes y Humanidades, Institución Universitaria de Barranquilla, Barranquilla 080003, Colombia; ∥ Colegio San José, Área de Ciencias Naturales, Puerto Colombia 081001, Colombia

**Keywords:** doped LaCrO_3_, refractory semiconductor, harsh environment conditions, band structure, ab initio calculations

## Abstract

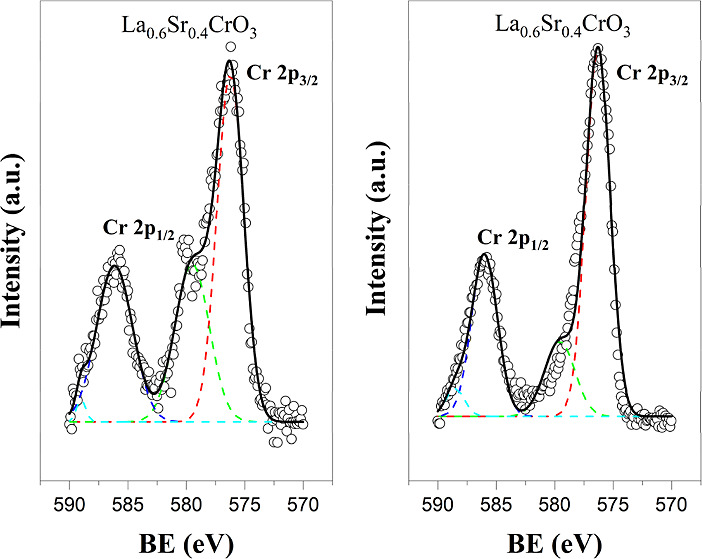

Doping effects on the valence state, electronic structure, and optical band and the effects on electrical conductivity were studied on the doped lanthanum chromite (LaCrO_3_) system. The specific compositions studied were La_1–*x*_Ca_*x*_CrO_3_ (LCC*x*), La_1–*x*_Sr_*x*_CrO_3_ (LSC*x*), and La_0.8_Sr_0.2_Cr_1–*x*_Mn_*x*_O_3_ (LSCM*x*) (0.1 ≤ *x* ≤ 0.4). The powders were synthesized using a modified Pechini sol–gel method, and the ceramic samples were densified using a reactive sintering method resulting in densities >96% theoretical. X-ray photoelectron spectroscopy (XPS) was completed to characterize the defect states and cationic valence compensation as a result of divalent (Ca^2+^ or Sr^2+^) and trivalent (Mn^3+^) substitutions. XPS was completed for samples tested in oxidizing and reducing atmospheres (up to 1500 °C), which provided insights into the oxidation state transitions induced by the Ca^2+^ and Sr^2+^ dopants. The work notably demonstrated, for the first time, the oxidation/reduction transitions of Cr^4+^ to Cr^3+^ in Sr^2+^/Mn^3+^ co-doped samples under reducing atmospheres. Reflectance UV–vis spectrophotometry optical band gap measurements were also completed for the same materials; a decrease in the optical band gap (2.81–3.12 eV) was shown with increased substitution, suggesting electronic structure modifications in the LaCrO_3_ perovskite. Density functional theory calculations validated experimental trends, predicting a diminishing band gap with a rising dopant concentration. The transition in Cr oxidation states was attributed to the presence of divalent/trivalent cations. These findings contribute some insights into methods to tune the LaCrO_3_ electrical properties for various low- and high-temperature applications.

## Introduction

Over the past few decades, researchers have extensively investigated perovskite LaCrO_3_ and its solid solutions formed through cation site doping. These materials have garnered significant attention due to their intriguing physical properties, including robust electrical conductivity,^1^ remarkable chemical and mechanical stability in both oxygen-rich and reductive environments,^2^ compatibility with other components in thermomechanical applications,^3^ high melting points,^4,5^ and wide band gaps (∼3.0 eV).^6^ Such properties position this composition as a promising candidate for various technological applications, including electrodes in solid oxide electrolysis cells,^7^ high-temperature electronics,^8^ harsh conditions sensing,^9^ electric heaters,^10^ energy storage,^11^ and chemical sensors.^12^ Its generalized perovskite composition of ABO_3_ allows it for versatile doping strategies, which includes adjustments to the mixed valence states and the semiconductor properties influenced by the dopant effects on the small polaron hopping mechanism and oxygen vacancy concentration.^6,13,19^ A-site doping with alkaline earth metals like Ca, Sr, Mg, and Ba,^14−17^ and B-site doping with transition metals such as Cu, Zn, Fe, Al, Co, and Mn, were explored to modulate the band gap, electrical conductivity, and thermoelectric properties (Seebeck coefficient).^18−21,42^

Studies on related systems have highlighted the critical role of cation substitution in tuning the optical, electronic, and structural properties of advanced materials. For instance, nanostructured powders of Sr_1.96_MgSi_2_O_7_ doped with Eu^2+^ and rare-earth codopants (e.g., Er^3+^, Tm^3+^, and Tb^3+^) demonstrated that the ionic radius of trivalent dopants significantly influences band gap energy and refractive index. Additionally, the combination of divalent activator Eu^2+^ with trivalent coactivators led to enhanced photoluminescence (PL) emission spectra, showcasing the interplay between the dopant size and emission properties.^22^ Similarly, codoping of Ca^2+^ and Ce^3+^ into Sn–ZnSn_2_O_5_-induced heterostructured morphologies, increased absorption in the visible light spectrum, and decreased optical band gap energy, demonstrating the synergistic effects of mixed-valence dopants in optimizing electronic and photoelectric conduction properties.^23^

In the same way, ZnSnO_3_ systems doped with combinations of Ca^2+^, Mn^2+^, and Cr^3+^ illustrated how multivalent doping can reduce band gap energy, modify lattice strain, and enhance optoelectronic responses, making these materials suitable for semiconductor and dielectric applications.^24^

On the other hand, as previously stated, one common dopant strategy for LaCrO_3_ includes the use of alkaline earths inserted into the A-site of the perovskite. Initial research started with the work published by Meadowcroft in 1969, where the electrical properties of the La_1–*x*_M^2+^_*x*_CrO_3_ system were investigated with incremental strontium substitution that was correlated with the electrical resistivity at temperatures >900 °C.^25^ The electrical conductivity mechanism was further studied over the past three decades for various other dopant strategies using divalent cation insertion into the A-site to control the suggested small polaron hopping conduction mechanism for this oxide composition.^26,27^ However, in the 1980s–90s, extensive research delved into tuning the electrical conductivity of LaCrO_3_. Studies such as Mori’s work^25^ highlighted that a 20% molar substitution of Ca^2+^ and Sr^2+^ lead to increased electrical conductivity in an air atmosphere (1.0–40.0 and 36.6 S cm^–1^ at 1000 °C, respectively). Conversely, under oxygen partial low pressures and H_2_ reducing atmospheres, electrical conductivity decreased due to the Cr^4+^ to Cr^3+^ transition and oxygen vacancy formation.^28^ Although doping the B-site with transition metal also improved the electrical conductivity,^29^ it was not until the 1990s that the conduction mechanism was experimentally determined to be small polaron hopping^30^ for dopants, such as Co and Mn. Taking into account the evidence that the substitution of La^3+^ (A-site) and/or Cr^3+^ (B-site) with divalent and/or trivalent cations, respectively, generates mixed valence states in LaCrO_3_, the need was raised to study in depth the modification of the band structure and the behavior of the electronic gap as a function of the dopant and its concentration.

Howng^31^ experimentally determined the band structure of LaCrO_3_ and the valence states in LaCrO_3_, (La, Sr)CrO_3_, and La(Cr,Mg)O_3_ systems by X-ray photoelectron spectroscopy (XPS). In this study, it was reported that a peak located at 575.8 eV corresponded to the Cr 2p_3/2_ state, which was related to Cr^3+^ and was identified in all three systems; however, when Sr^2+^ and Mg^3+^ were introduced, the peak broadening indicated the formation of Cr^4+^, giving evidence of the polaron hopping conductive mechanism. In the same way, the authors reported the charge-transfer transition 2p → 3d observed by a new peak formation at ∼581.5 eV related to the formation of Cr^6+^. Similar observations in the (La, Sr)CrO_3_ system were reported by other authors.^32^ The mixed valence states of the (La, Ca)CrO_3_ system was also investigated and the effects of Ca^2+^ at different molar concentrations of *x* = 0.10,^34^ 0.20,^35^ 0.32,^36^ 0.50,^33^ and 0.60.^34^ In these works, a similar electronic hole formation and polaron hopping mechanism was assumed because the XPS data showed a change in the intensity of the Cr 2p_3/2_ peak. This change again was mainly determined by the contributions of the oxidation states Cr^3+^ and Cr^4+^, which were assigned to this peak. The mixed valence states were also studied for other doped lanthanum chromite systems such as (La, Ce)CrO_3_,^37^ (La, Ag)CrO_3_,^38^ and La(Cr,Pd)O_3_,^39^ which exhibited the same Cr state behavior. However, all mentioned studies were performed under a normal air atmosphere; there was no significant literature reporting experimental studies on the mixed valence state transitions of doped-LaCrO_3_ under reducing conditions.

The optical band gap measurements can also provide complementary information to predict the electrical conductivity and potential modification of the doped chromite band structures. A few authors have completed studies based on the effects of dopants (and their concentrations) on the optical band gap trends. Polat reported the band gap modification in LaCr_1–*x*_M_*x*_CrO_3_ (where M = Co, Pd, Ir) as a function of the substitute content. They observed that by increasing the metal content, the optical band gap decreased relative to pure LaCrO_3_ (*E*_gap_ = 3.39 eV^40^), where the lowest values were 2.93, 2.69, and 2.66 eV for Pt, Co, and Ir, respectively. This band gap decrease behavior was also shown in other studies, where LaCrO_3_ was doped with other transition metals, such as Zn^41^ and Fe,^42^ producing an optical band gap drop from 3.39 eV (for the pure LaCrO_3_ composition) to 1.65 and 2.91 eV, respectively. However, there are no literature reports that correlate the optical band gap trends as a function of the divalent dopant concentration of Ca^2+^ and Sr^2+^.

Different attempts were published over the past decade to determine the band structure of LaCrO_3_ and various doped variances using ab initio calculations. These calculations enable the construction of a comprehensive model of the electronic band structure of these materials. These works focused on the general perovskite LaMO_3_ (M = Fe, Cu, Ti, Cr, Mn) systems using density-function theory [density functional theory (DFT)] calculations.^43−48^ In these works, the structural, magnetic properties, and band structure of pure LaCrO_3_ were estimated. The DFT modeling results showed a G-type antiferromagnetic behavior with an optical gap of 3.1 eV; the valence band states were mainly contributed by Cr–3d (t_2g_^3^) and O–2p electrons with small contributions of La–5d electrons, while the conduction band minimum states were Cr–3d (t_g_^0^).^46,47^ In the work published by Yu,^49^ the authors determined the band structure of pure LaCrO_3_, Ca–LaCrO_3_, and Ca/Fe–LaCrO_3_ by DFT modeling and observed that the introduction of Ca and Fe in the LaCrO_3_ structure produced the formation of new defect states above the valence band maximum, decreasing the pure LaCrO_3_ optical band gap from 3.1 to 2.33 and 2.44 eV, respectively.^49^ Unfortunately, the contribution of oxygen vacancies on the band structure and optical properties were not considered in this modeling. However, Deml^54^ developed a theoretical approach using the introduction of oxygen vacancies in their calculations. The authors found exclusively for the system (La, Sr)CrO_3_ that the density of states displayed defects related to the Cr^3+^ → Cr^4+^ transition and demonstrated theoretically with more accuracy the experimentally observed p-type electronic transport by small polaron hopping.^50^ More recent studies have successfully applied both experimental methods and DFT to investigate the electronic, structural, and optical properties of Co-doped LaCrO_3_. For instance, Xu.^51^ demonstrated that Co-doping in LaCrO_3_ significantly reduces the band gap and modifies the band structure by introducing impurity levels near the Fermi level, enhancing optical absorption. These insights highlight the role of dopants in controlling electronic transitions and lattice deformations, which directly influence the band structure, a key aspect further explored in this work.

The current work seeks to build upon the insights gained from previous research studying the defect evolution under various atmospheric conditions and its effect on the band structure for various doped LaCrO_3_ compositions. The current study particularly focuses on Ca, Sr, and Sr/Mn dopants. Previous studies have investigated the valence states using XPS and the optical band gap using UV–vis spectroscopy, but as noticed, they often lack consistency in methodology in relation with synthesis (including solid-state reactions and wet methods), annealing conditions (only under air atmosphere), and dopant and multiple concentration levels (any work report more than three levels). Again, it must be pointed out that the current work employs a uniform synthesis and processing method for all compositions to better compare results between these compositional systems. It was found to be difficult to compare the results over various authors over the past few decades because each publication used different methodologies to form singular doped LaCrO_3_ ceramics, with each having a different density, grain size, and dopant distribution. Additionally, this work incorporates DFT modeling for all dopant systems, providing a more accurate representation of the electronic band structure. Moreover, the analysis is extended beyond theoretical calculations by comparing optical band gap values obtained from DFT modeling to experimental measurements. This comprehensive approach allows us to assess the synergy between experimental and theoretical results and elucidate the underlying mechanisms governing the electrical properties of LaCrO_3_-doped materials, providing solid scientific foundations for future investigations and technological applications in this field.

## Experimental Section

### Lanthanum Chromite Ceramic Fabrication Method

The Ca, Sr, and Sr/Mn-substituted lanthanum chromites (La_1–*x*_Ca_*x*_CrO_3_, La_1–*x*_Sr_*x*_CrO_3_, and La_0.8_Sr_0.2_Cr_1–*x*_ Mn_*x*_O_3_ (0.1 ≤ *x* ≤ 0.4)) compositions were synthesized by a Pechini sol–gel method employing citric acid (HO_2_CCH_2_–C(OH)(CO_2_H)CH_2_CO_2_H) (99.9% purity) and La(NO_3_)_3_·6H_2_O (99.9% purity), Cr(NO_3_)_3_·9H_2_O (99.0% purity), Sr(NO_3_)_2_ (99.5% purity), Ca(NO_3_)_2_ (99.5% purity), and Mn(NO_3_)_2_·*x*H_2_O (99.0% purity) as metal sources (from Sigma-Aldrich, USA). To simplify the nomenclature through this paper, compositions will be labeled as follows: LCC*x*, LSC*x*, and LSCM*x* for Ca, Sr, and Sr/Mn substitution, respectively, where *x* indicates the percentage molar substitution level (10–40%). All metal nitrates were dissolved in deionized water, and the accuracy of the final metal concentrations was established by an inductively coupled plasma-mass spectrometer (ICP-MSPerkin Elmer NexION 2000, USA) following EPA Method 200.8. The required solution concentration of metal species for each studied composition was weighed and mixed with citric acid at an equivalent molar ratio of 1:2 (metal cations/citric acid). The solutions were heated to 80 °C to form a viscous gel which yielded a purple solid precursor during slow drying. The dried gel was then thermally treated at 400 °C for 1 h to remove the organic material. Samples were pulverized manually in a mortar and calcined at 900 °C for 5 h, where the perovskite phase was not formed yet at this temperature, and the fine particle size of the precursor was retained. These uncrystallized powders were then suspended in isopropyl alcohol (>99%) and milled on a ball rolling system (US Stoneware 755RMV) using 3 mm ZrO_2_-milling media for 2 h. Dense polycrystalline pellets of the powder were formed by initially uniaxially pressing under 1 MPa of pressure into circular pellets 50 mm in diameter and approximately 5 mm in thickness. The green bodies were sintered at 1600 °C with a heating rate of 2 °C/min under dry air with an isothermal holding time of 2 h, where a “reactive sintering” process was carried out, which refers to the concurrent occurrence of full perovskite phase formation during calcination and the sintering mechanism. This approach focuses on maintaining the small particle size of the precursor materials to ensure densification by a high driving force. The sintered samples’ theoretical density was about 98%. To analyze the valence state of ions in LCC*x*, LSC*x*, and LSCM*x* compositions under various atmospheres by XPS, polished pellets were annealed at 1300 °C for 5 h in oxidizing (dry air) and reductive atmospheres (forming gas H_2_/N_2_ 5%: 95%).

### Physical and Electrical Characterization Studies of Doped Lanthanum Chromite Ceramics

The phase purity, successful doping, and/or uniform elemental incorporation within the lattice for all synthesized compositions were rigorously confirmed using X-ray diffraction (XRD, PANalytical X’pert PRO, Cu Kα radiation, model number PW 3040 Pro). Power requirements during the operation were 45 kV and 40 mA, respectively. The divergence slit angle for the incident X-ray beam was set to 0.5°. Scans were performed with a 0.033°/s scan rate and a 40 s step time. More detailed scans were performed by using a 0.016°/s scan rate and 400 s step time. An X’Pert High Score software was used to identify the phases and crystal structure. Detailed XRD patterns and further validation of successful doping are provided in the Supporting Information section for further reference. XPS measurements were also carried out to determine changes in mixed valence states and binding energy of the metallic constituent elements present in all samples after annealing under oxygen-rich, inert, and reducing atmosphere conditions. Polished pellets were analyzed using a Physical Electronics, PHI 5000 Versa Probe spectrometer with a monochromatic Al Kα source operated at 300 W and a base pressure of 5 × 10^–8^ Torr at room temperature. As a reference, the C 1s signal of the adventitious carbon was used, which was fixed at 284.6 eV. Survey spectra were collected by 1.0 eV steps at an analyzer pass energy of 160 eV and the high-resolution analysis of small spectrum regions by 0.05 eV steps and a pass energy of 20 eV. The composition and chemical states were determined from the charge-corrected high-resolution scans with an analyzer pass energy of 20 eV. The acquisition time of the sample was kept low to minimize any surface oxidation state changes under X-ray irradiation. All spectra were fitted with a Shirley background, and a Voigt function was employed to simulate spectral lines with a Gaussian component set to 40% for spectra collected with the Kratos instrument and set to 70% for the PHI and SSX instruments. The best fits were determined by minimization of the summed root-mean-square value of the fit. Atomic percentages were calculated from survey spectra of all sample surfaces from peak area ratios normalized by the appropriate atomic sensitivity factors.^52^ To study the optical band gap trends over all the polished pellet samples, diffusive reflectance spectrophotometry measurements were carried out through a Shimadzu 2600 UV–vis spectrophotometer (Shimadzu Corporation, Kyoto, Japan). The spectra were acquired in the 330–600 nm range for all samples using barium sulfate as a verification standard.

### Band Structure, Density of States, and Optical Band Gap Modeling Using Density Functional Theory

**Figure 1:**
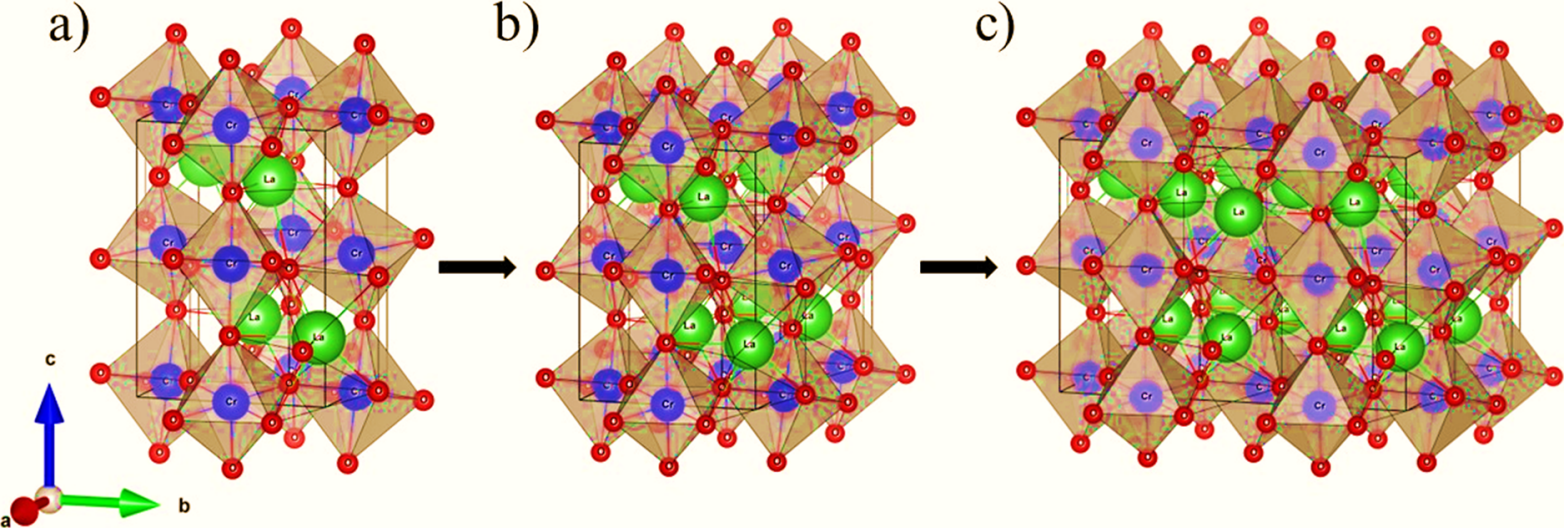
DFT modeled supercell of the LaCrO_3_: (a) unit cell, (b) 2a × 1b × 1c, and (c) 2a × 2b × 1c.

Spin-polarized first-principles calculations were carried out within the framework of DFT. The exchange and correlation effects were addressed using the generalized gradient approximation (GGA) and the GGA + *U* approach, as implemented in the Perdew–Burke–Ernzerhof (PBE) functional.^53^ Effective parameters (*U*_eff_) of 3.3 and 6.0 eV were applied to the 3d orbitals of Cr and Mn atoms, respectively, in line with values reported in ref (54,55,) which accurately described the bulk electronic structure of both pure and doped lanthanum chromite. These parameters were adopted for the calculations in this study. The plane-wave projector-augmented wave method^56,57^ was utilized as incorporated in the Vienna Ab initio simulation package (VASP).^58,59^ The electron wave functions were expanded by using plane waves with a cutoff energy of 520 eV. *K*-point meshes of 3 × 6 × 4 and 3 × 3 × 4 were applied for supercells of 2a × 1b × 1c and 2a × 2b × 1c, based on a conventional LaCrO_3_ (ABO_3_) orthorhombic unit cell (see Figure 1). Substitutions were modeled by replacing two (one), four (two), and six (three) La or Cr atoms with A^2+^ = Sr, Ca^2+^ or B^3+^ = Mn atoms, respectively, to achieve substitution concentrations of *x* = 0.125 (12.5%), *x* = 0.250 (25%), and *x* = 0.375 (37.5%) in La_1–*x*_Sr_*x*_CrO_3_ (LCC*x*), La_1–*x*_Ca_*x*_CrO_3_ (LSC*x*), and La_0.8_Sr_0.2_Cr_1–*x*_Mn_*x*_O_3_ (LSCM*x*). For LaCrO_3_, the ground state was determined to be G-type antiferromagnetic ionization (G-AFM). Total energy calculations were performed for the ferromagnetic (FM) state as well as three antiferromagnetic configurations (A-AFM, C-AFM, and G-AFM) with optimized atomic positions, which are consistent with previous experimental and theoretical findings.^46^

## Results and Discussion

### XPS Characterization of Valence States

**Figure 2:**
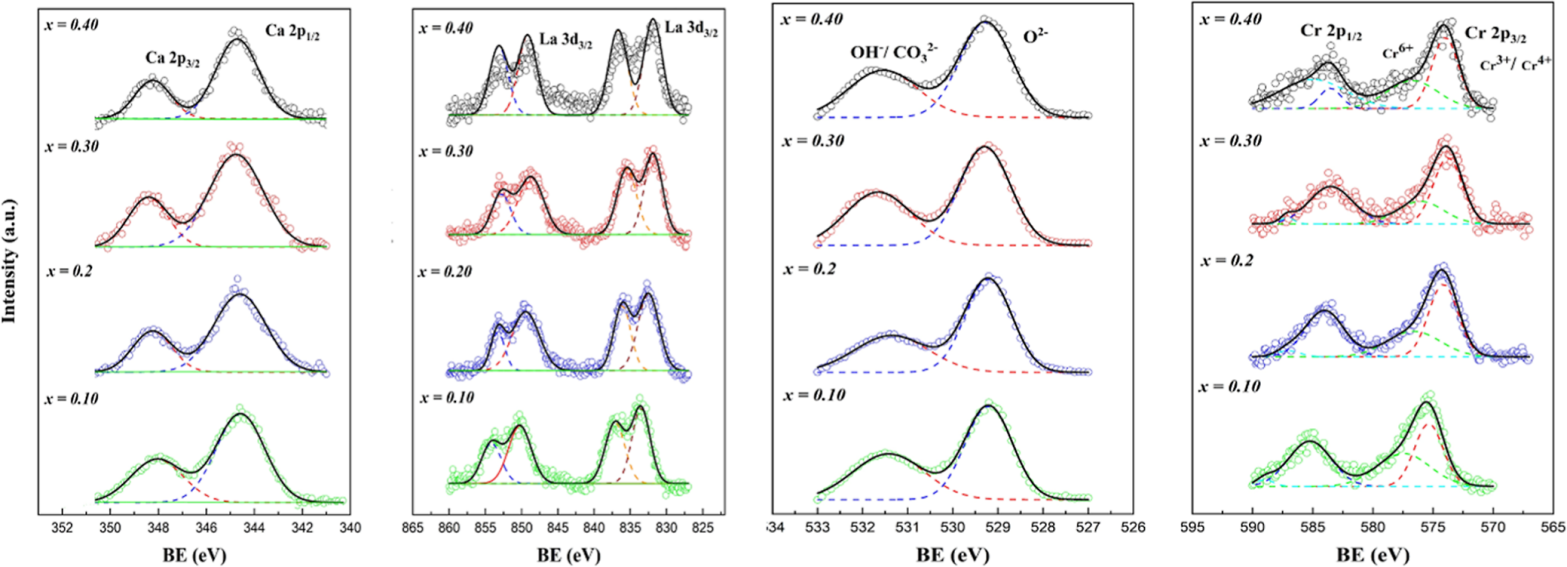
Ca 2p, La 3d, O 1s, and Cr 2p core level XPS spectra and representative fittings of LCC*x* compositions annealed under an oxidizing atmosphere for 5 h.

The LCC*x*, LSC*x*, and LSCM*x* compositional systems were annealed in both oxidizing and reducing atmospheres and characterized by XPS to define the evolution of the defect states of these materials. Figure 2 shows the Ca 2p, La 3d, Cr 2p, and O 1s core level XPS spectra and representative fittings of LCC*x* compositions annealed under a dry air atmosphere (oxidizing condition) for 5 h. It can be observed that by introducing and systematically increasing the Ca^2+^ content between *x* = 0.1–0.4, no significant binding energy shifts were seen for the La 3d region. Peaks located at 831 and 837 eV of La 3d_5/2_ and those at 847.5 and 852.5 eV of La 3d_3/2_ were detected and related to La^3+^ cation in the oxide form.^60,61^ Two peaks for Ca 2p were detected at 345 and 348 eV for Ca 2p_3/2_ and Ca 2p_1/2_, respectively, without significant shifts. This suggests the chemical stability of the calcium in the doped lanthanum chromite perovskite structure. Increasing the calcium content did not affect the oxygen valence states; significant changes were not detected for the two observed peaks in the O 1p zone. The peak at 526.4 eV is related to lattice oxygen, while a broader peak in 529 eV can be assigned to chemisorbed oxygen in the form of OH^–^ and CO_3_^2–^.^33,62^ In the area of Cr 2p, a peak located at 576 eV corresponds to Cr 2p_3/2_ associated with the presence of Cr^3+^ and Cr^4+^.^65^ A quantitative analysis is difficult for these valence states because both species appear at similar binding energies, 575.7 eV (Cr^4+^) and 576.1 eV (Cr^3+^).^37,63^ As mentioned before, LaCrO_3_ is a p-type semiconductor, and when Ca^2+^ is introduced in the A-site, then the Cr^3+^ (3d^3^) oxidizes to Cr^4+^ (3d^2^) creating electronic holes. However, under oxidizing conditions, the Cr^4+^ can be also oxidized to Cr^6+^ (3d^0^) which can form CrO_3_ which may volatilize and/or be deposited on the surface of the material at high temperatures.^35^ The peak located at 579 eV is related to Cr^6+^.^35,37,63^ It can be observed that by increasing the Ca^2+^ content, the intensity of these peaks increased proportionally. As mentioned earlier, introducing a divalent acceptor dopant, such as Ca^2+^ into the La^3+^ A-site, necessitates maintenance of the perovskite electroneutrality. This requirement implies that the effective charge of the substituent cation must be compensated through either electronic or ionic means, leading to the oxidative transition of Cr^3+^ or the formation of oxygen vacancies. Kröger–Vink notation represents this condition as follows
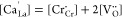
1where the concentration of enclosed species is represented by brackets ([]), with a single prime denoting a unit negative charge related to the host lattice, *V* representing oxygen vacancies, and superscripted dot indicating a unit positive charge concerning the lattice. Under an air atmosphere, the oxygen content on the divalent doped LaCrO_3_ is stoichiometric and the electronic compensation leads to an elevation in the valence of chromium, suggesting that the compensation is led by an electronic mechanism and eq 1 can be expressed as
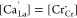
2

Table 1 summarizes the binding energies of the core electrons peaks of the XPS spectra for La 3d, Ca 2p, Cr 2p, and O 1s of all LCC*x* compositions, including the analysis of Figure 1 for samples annealed in oxidizing conditions, as well as the unshown data of peak shifts for samples annealed under a reducing atmosphere. The table also displays the shifts in binding energies of the main peaks; overall, it is observed that the binding energies of La 3d_5/2_, Ca 2p_3/2_, and O 1s show no consistent variation with the change in the Ca concentration in the samples. As the calcium concentration increases from LCC10 to LCC40, there is a general trend of increasing binding energies for Cr 2p_3/2_. This suggests a significant influence of calcium doping on the bonding properties inside LaCrO_3_ materials, especially in relation to Cr atoms, particularly in an oxidizing atmosphere. The observations are in agreement with refs (34,35). On the other hand, according to Table 1, under a reducing atmosphere, the characteristic La 3d_5/2_, Ca 2p_3/2_, and O 1s binding energy peaks showed smaller shifts, indicating no significant change in the chemical environment for these atoms, as well as the shifts observed under oxidizing conditions. It was observed that for Cr 2p_3/2_, the peaks were located at ∼572 and 576 eV for Cr^3+^/Cr^4+^ and Cr^6+^, respectively. Under reducing conditions, the transition Cr^6+^ → Cr^4+^ → Cr^3+^ occurs producing a decrease in the hole concentration and oxygen vacancy formation, activating in this way the ionic conduction mechanism.^28^

**Table 1:** Binding Energies of Core Electron Peaks from XPS Spectra of LCC*x*, Annealed under Oxidizing (Dry Air) and Reducing Atmospheres (Forming Gas H_2/_N_2_ 5%: 95%)

	XPS binding energies of main peaks (eV)									
	oxidizing atmosphere					reducing atmosphere				
sample	La 3d_5/2_	Cr 2p_3/2_	Ca 2p_1/2_	Ca 2p_3/2_	O 1s O^2–^	La 3d_5/2_	Cr 2p_3/2_	Ca 2p_1/2_	Ca 2p_3/2_	O 1s O^2–^
LCC10	833.57	573.96	345.30	348.20	530.03	830.20	572.04	343.50	348.11	527.76
	836.97	577.13				834.48	576.12			
LCC20	832.57	573.86	344.61	348.12	529.04	830.45	572.11	343.87	347.93	527.72
	835.99	576.24				834.66	576.32			
LCC30	831.88	574.36	344.82	348.00	529.08	830.27	572.49	343.42	348.21	527.89
	835.46	576.63				834.37	576.49			
LCC40	832.27	575.35	345.77	347.94	530.27	830.89	573.56	344.01	348.06	527.23
	836.06	577.43				834.76	576.66			

Table 2 shows the summary of the XPS atomic percent content of the doped LaCrO_3_ elemental constituents after being annealed under oxidizing and reducing atmospheres. For LCC*x*, the relative atomic percent for La^3+^ remained stable under both oxidizing and reducing conditions for all compositions. The percentage of Ca^2+^ increased proportionally with the substitution level and remained stable in relation to the working atmosphere. Significant changes in transitions can be observed for the Cr and O contents. In all cases, Cr^3+^/Cr^4+^ increased proportionally to the Ca^2+^ content. A smaller proportion of Cr^6+^ was also present due to the formation of CrO_3_ under the oxidizing conditions. However, under the H_2_/N_2_ atmosphere, Cr^6+^ content decreased and the concentration for Cr^3+^/Cr^4+^ increased, indicating the increase of Cr^3+^. As discussed before, to achieve charge neutrality, oxygen vacancies should be formed under reducing conditions. It can be observed in Table 2 that the lattice oxygen concentration decreased; the evidence of oxygen vacancy formation by XPS analysis was also observed by a previous author.^64^ XPS confirmed the formation of oxygen vacancies and mixed valence states predominantly near the surface, particularly under reducing conditions. The high annealing temperatures (1300 °C) and extended holding times (5 h) promote diffusion-driven processes that facilitate the migration of defects from the surface into the bulk. This defect migration plays a crucial role in creating localized electronic states that enhance charge transport through the activation of the small polaron hopping mechanism, as evidenced by DC electrical conductivity measurements provided in the Supporting Information section.

**Table 2:** Summary of the XPS Surface Analysis Percent Concentration of Metal Cations^a^

	XPS analysis (%)								
composition	La^3+^	Cr^3+^/Cr^4+^	Cr^6+^	O^2–^	Ca^2+^	Sr^3+^	Mn^3+^	Mn^4+^	Mn^2+^
LCC10 oa	15.1	17.4	1.8	62.6	3.1				
LCC10 ra	18.8	18.3	0.5	56.1	3.3				
LCC20 oa	15.3	18.5	1.7	62.4	4.1				
LCC20 ra	19.4	19.2	0.5	56.4	3.8				
LCC30 oa	13.4	21.8	1.7	61.8	6.3				
LCC30 ra	19.4	22.7	0.6	55.8	5.9				
LCC40 oa	11.9	24.3	1.6	62.6	7.6				
LCC40 ra	19.2	25.7	0.5	54.6	7.4				
LSC10 oa	16.4	18.7	1.6	62.9		3.4			
LSC10 ra	19.9	20.5	0.4	60.8		2.4			
LSC20 oa	15.4	21.2	1.6	62.1		4.9			
LSC20 ra	18.9	23.1	0.3	59.6		4.1			
LSC30 oa	13.7	23.3	1.5	62.8		6.7			
LSC30 ra	17.7	25.4	0.4	58.0		6.5			
LSC40 oa	12.8	14.9	1.5	62.6		8.2			
LSC40 ra	15.1	27.4	0.5	57.6		8.4			
LSCM10 oa	16.4	29.5	1.6	61.2		2.8	2.0	0.6	
LSCM10 ra	19.5	16.0	0.8	59.4		2.3	1.2	0.8	
LSCM20 oa	16.7	13.1	1.3	62.4		2.6	3.1	0.8	
LSCM20 ra	20.5	12.9	0.8	59.6		2.8	2.3	1.1	
LSCM30 oa	15.9	12.4	1.3	61.8		3.2	4.9	0.3	1.0
LSCM30 ra	19.8	13.1	0.7	57.8		2.8	4.1	0.2	1.6
LSCM40 oa	16.7	11.4	1.2	60.6		2.7	5.8	0.6	0.8
LSCM40 ra	19.5	11.8	0.6	58.2		2.8	5.0	0.2	1.8

a (oa: oxidizing atmosphere; ra: reducing atmosphere).

**Figure 3:**
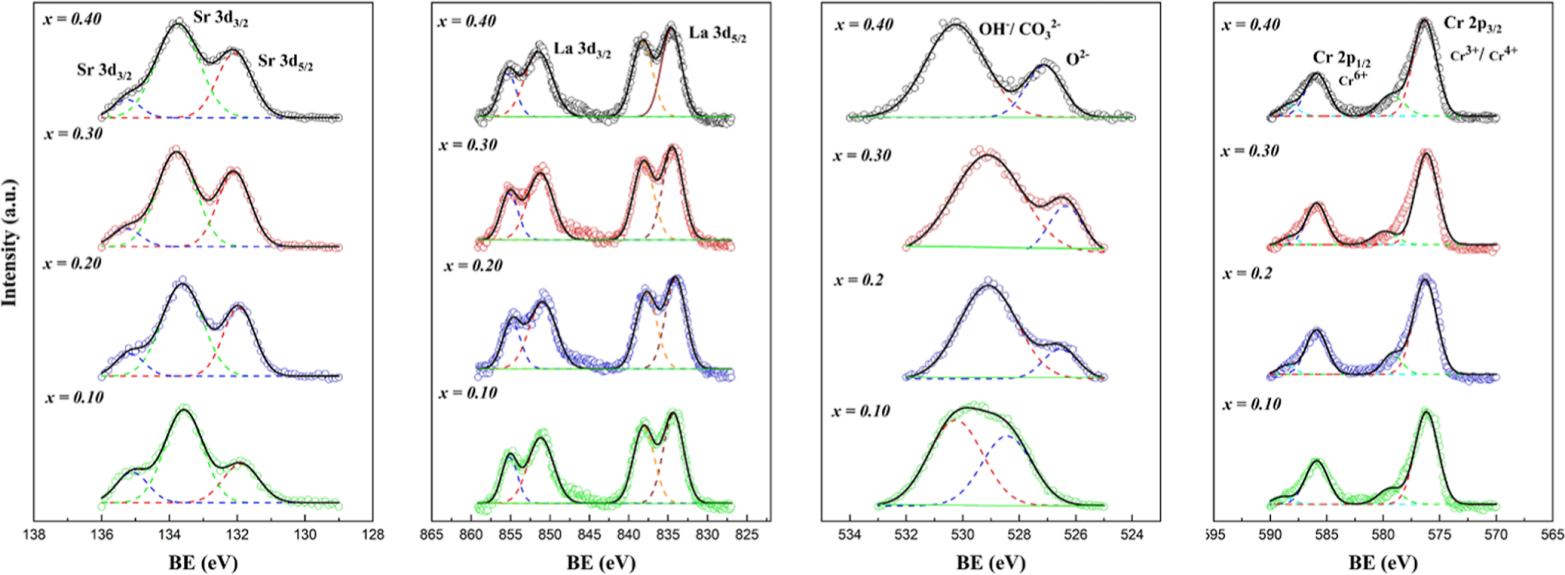
Sr 3d, La 3d, O 1s, and Cr 2p core level XPS spectra and representative fittings of LSC*x* compositions annealed under reducing atmospheres for 5 h.

**Figure 4:**
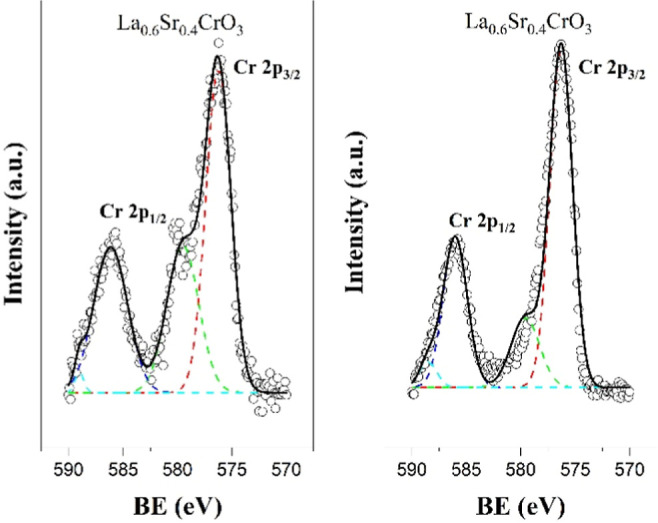
Oxidizing (left) to reducing (right) atmosphere transition for Cr 2p_1/2_ (Cr^6+^) peak at 579 eV in LSC40.

Figure 3 shows the Sr 3d, La 3d, Cr 2p, and O 1s core level XPS spectra and peak fittings of the LSC*x* compositions annealed under reducing atmospheres for 5 h. Under the reductive atmosphere, the binding energy peaks at 833.85 and 838.64 eV for La 3d (La^3+^) and 133.36 eV for Sr 3d (Sr^2+^) did not show any shift for the different strontium doping levels compared to the oxidizing conditions. For the O 1s, lattice oxygen peaks at ∼526.00 eV were detected with a binding energy shift of 2 eV in contrast with oxidizing conditions. For Cr 2p_3/2_, it was observed that a change in the Cr^3+^/Cr^6+^ ratio from 2.35 to 1.58; the Cr^3+^/Cr^4+^ peak was more predominant in the Cr 2p_3/2_ area. A decrease in the Cr^6+^ peak intensity was noticeable for all of the LSC*x* compositions. Figure 4 shows the oxidizing to reducing atmosphere transition in the Cr 2p_1/2_ (Cr^6+^) peak at 579 eV in LSC40 indicating the reduction process of Cr^6+^ → Cr^4+^ → Cr^3+^. In order to keep the charge neutrality of the compound, oxygen vacancies were formed in the system, and the polaron hopping conduction mechanism was less active, as discussed for the LCC*x* systems.

Table 3 shows the binding energies of core electron peaks from the XPS spectra of LSC*x*, annealed under oxidizing and reducing atmospheres. The binding energy data were used to determine the valence states of the ions in the structure under oxidizing atmosphere conditions. The peaks located at 834.38 and 838.20 eV are related to La 3d_5/2_ and indicate that the ion state is La^3+^. An increase in the strontium substitution concentration did not affect the chemical environment of lanthanum. The binding energy values of Sr 3d_5/2_ are related to the Sr^2+^ state, although the location of the observed peak remained constant. It was observed that at temperatures up to 1200 °C, secondary phases of strontium chromate (SrCrO_4_) can be formed due to the low solubility of Sr^2+^ under these conditions;^65^ however, the binding energy of strontium in the chromite, chromate, and oxide forms are similar to binding energies of 133.0 eV for the first two and 133.40 for the last, making it difficult to relate the cation to a specific compound using XPS.^32^ In the same way, as for the LCC*x* compositions, a lattice oxygen O 1p peak is observed at 529.0 eV and a second at 531 eV, which was assigned to chemisorbed oxygen in the form of OH^–^ and CO_3_^2–^, as previously mentioned. Also, when the Sr^2+^ is introduced in the lanthanum chromite structure, it produces changes in the valence states of Cr^3+^, oxidizing to Cr^4+^ to maintain charge neutrality; however, Cr^6+^ is also formed in the transition. The Cr 2p peak located around 576.1 eV is related to the mixed valence Cr^3+^/Cr^4+^; the binding energies of both ions are not easily distinguished.^63^ A second peak at ∼579 eV is related to Cr^6+^. It was observed that increasing the content of strontium produced the broadening of this peak, displaying the formation and increase in the hole concentration due to Cr^3+^ → Cr^4+^ → Cr^6+^.

**Table 3:** Binding Energies of Core Electrons Peaks from XPS Spectra of LSC*x*, Annealed under Oxidizing and Reducing Atmospheres

	XPS binding energies of main peaks (eV)									
	oxidizing atmosphere					reducing atmosphere				
sample	La 3d_5/2_	Cr 2p_3/2_	Sr 3d_5/2_	Sr 3d_3/2_	O 1s O^2–^	La 3d_5/2_	Cr 2p_3/2_	Sr 3d_5/2_	Sr 3d_3/2_	O 1s O^2–^
LSC10	834.38	575.71	131.94	133.52	528.02	833.85	575.87	131.96	133.58	528.17
	838.20	576.98				838.64	579.01			
LSC20	834.41	575.86	131.96	133.61	527.78	834.07	576.20	131.86	133.60	526.13
	838.21	577.39				838.00	579.20			
LSC30	834.38	575.92	131.87	133.68	527.96	833.98	575.46	131.93	133.59	525.92
	838.16	578.78				838.10	576.10			
LSC40	834.70	576.28	131.88	133.71	528.00	833.91	575.81	131.84	133.64	526.24
	838.20	579.80				838.25	576.23			

In a manner similar to the characterization performed for the LCC*x* compositions, the atomic percentage of the elemental constituents of LSC*x* is determined and summarized in Table 2. This comprehensive analysis allows for the identification of concentration changes, particularly for Sr^2+^, indicating a proportional percentage increase corresponding to the substitution level. Furthermore, the concentration of Cr^3+^/Cr^4+^ increased proportionally with the Sr^2+^ content. Notably, the presence of a small concentration of Cr^6+^ suggests the formation of CrO_3_. Interestingly, the percentage of Cr^6+^ decreased under the reducing atmosphere, accompanied by a notable increase in the concentration of Cr^3+^, indicating a dynamic response to environmental conditions. These findings provide compelling evidence that for divalent substitution on the La^3+^ site, the oxidation state of Cr^4+^ plays a pivotal role in defining the polaron hopping mechanism. Additionally, the observation of secondary phases, such as CrO_3_ and oxygen vacancies, under reducing conditions underscores the complex interplay between dopant species and environmental factors. This detailed analysis not only contributes to the understanding of the structural and compositional evolution of doped LaCrO_3_ but also aligns closely with our objectives to provide a comprehensive electrical characterization of these materials.

This study represents the first comprehensive characterization of mixed valence states in the Sr–Mn-doped LaCrO_3_ (LSCM*x*) system. This is important because the understanding of valence transitions and their impact on electrical properties in various atmospheres for this composition is pivotal to its application as an anode for the solid oxide fuel cell (SOFCs)^66^ or as a cathode for electrolysis cells (SOECs),^67^ due to its chemical stability under reducing conditions and sulfur poisoning and coking^68^ as an anode material. In this context, LSCM*x* stands out as a viable option due to its dual oxygen ion and electronic conductivity, positioning it as a potential electrode material for high-temperature electrical applications^69^ and addressing the critical need for materials with enhanced performance and stability in reduced SOFC operating conditions.

**Figure 5:**
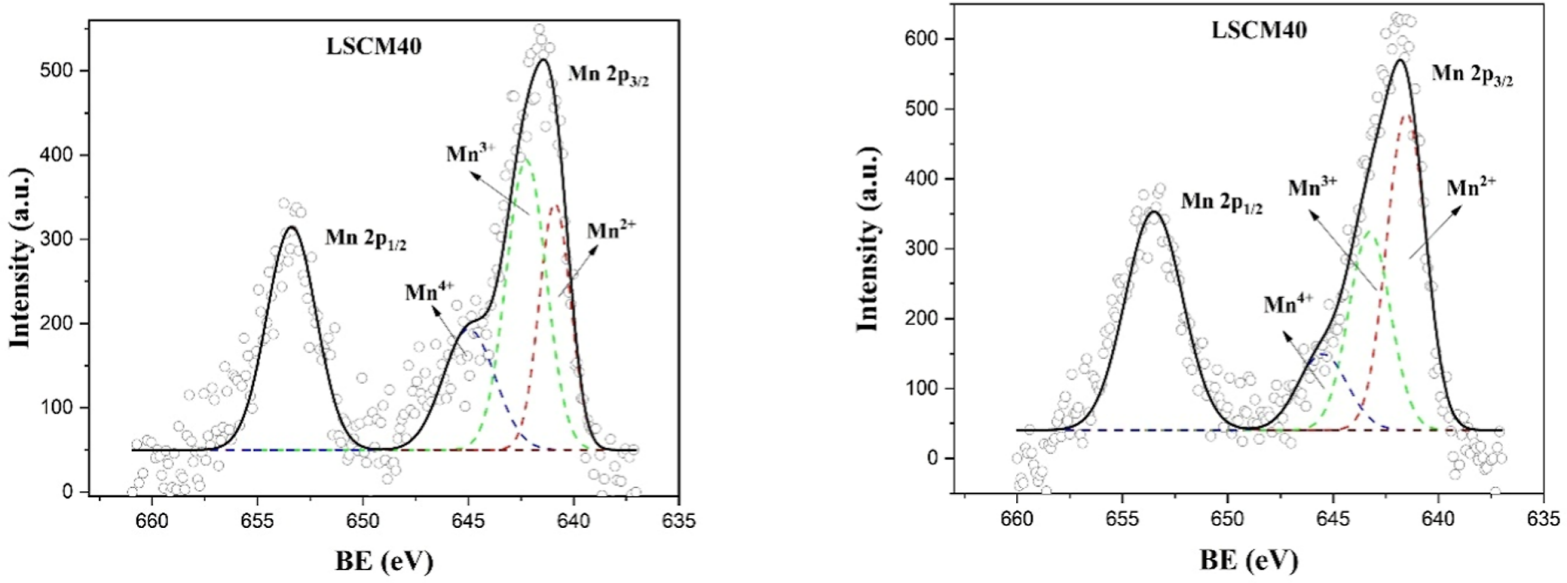
Oxidizing (left) to reducing (right) atmosphere transition for Mn 2p_3/2_ (Mn^2+^, Mn^3+^, and Mn^4+^).

In the same way as the divalent cation-doped LaCrO_3_ mixed valence state discussions, Table 4 shows the binding energies of core electron peaks from XPS spectra of LSCM*x*, annealed under oxidizing and reducing atmospheres. As observed in LSC*x* compositions under both oxidizing and reducing atmospheres, the peaks related to the La 3d_5/2_ were located at ∼834.00 and ∼838.00 eV, which indicates the La^3+^ state. For the Sr 3d_5/2_ zone, the binding energy peak at ∼133.00 eV indicates the Sr^2+^ state. In the same way, the peak at 529.00 eV corresponds to the lattice oxygen, as observed before in LCC*x* and LSC*x*. However, a small shift of this peak between the oxidizing and reducing atmospheres suggests the formation and segregation of MnO during the reduction transition. No significant binding energy shifts were observed for these ion valence states due to the changing of the working atmosphere condition, indicating chemical stability and no secondary phase formation. Valence states for chromium were Cr^3+^/Cr^4+^ with a peak located at 576.00 eV and Cr^6+^ peak near 579.00 eV in both atmospheres. As with the LCC*x* and LSC*x* compositions, a change in the ratio Cr^6+^/Cr^3+^ was observed from oxidizing to reducing atmospheres, indicating the reducing transition Cr^3+^ → Cr^4+^ → Cr^6+^. Three manganese valence states were observed; a peak at 642.00 eV indicates the Mn^3+^ state, as expected for the substitution of Cr^3+^; however, the introduction of a divalent cation in the A-site (Sr^2+^), produced the oxidation of Mn^3+^ to Mn^4+^. The presence of this state is related to the peak at 645.00 eV as observed for all the manganese substitution levels under oxidizing conditions. However, increasing the manganese content to 30% and 40% produced the formation of Mn^2+^ with a peak located at 641.00 eV, possibly related to the formation of a manganese oxide secondary phase. Under the reducing atmosphere, all three binding energy valence states were observed in the same regions. The intensity and ratio changed as supported by the cation percentage concentration shown in Table 2. The Mn^2+^ content was shown to increase while the Mn^4+^ content decreased. Figure 5 shows the oxidizing to reducing atmosphere transition for Mn 2p_3/2_: Mn^4+^ → Mn^3+^ → Mn^2+^, where clearly the peak ratio changed for the LSCM40 composition. This is a similar result that was shown for the LSCM30. Table 2 also shows that for the LSCM*x* compositions the concentration of La^3+^ and Sr^2+^ remains without a significant variation in relation to both oxidizing and reducing conditions. The Cr^3+^/Cr^4+^ and Cr^6+^ concentration trends were similar to that observed for the LSC*x* compositions.

**Table 4:** Binding Energies of Core Electron Peaks from XPS Spectra of LSCM*x*, Annealed under Oxidizing and Reducing Atmospheres

	XPS binding energies of main peaks (eV)									
	oxidizing atmosphere					reducing atmosphere				
sample	La 3d_5/2_	Cr 2p_3/2_	Sr 3d_5/2_	O 1s O^2–^	Mn 2p_3/2_	La 3d_5/2_	Cr 2p_3/2_	Sr 3d_5/2_	O 1s O^2–^	Mn 2p_3/2_
LSCM10	834.27	576.12	133.36	529.67	642.80	834.48	576.28	133.97	529.71	641.75
	838.34	579.03			645.12	838.74	578.89			
LSCM20	834.15	576.22	133.53	529.58	641.93	834.37	576.32	133.84	529.23	641.71
	838.63	578.94			645.68	838.74	579.01			642.35
LSCM30	834.48	576.12	133.65	529.08	641.51	834.65	576.34	133.59	529.66	641.68
	838.42	578.45			642.48	838.28	579.32			642.44
					645.19					
LSCM40	834.79	576.33	133.58	529.37	641.29	834.55	576.43	133.72	529.31	641.32
	838.74	579.44			642.90	838.71	579.19			642.49
					644.96					

### Band Gap Determination by UV/Vis Diffuse Reflectance Spectra

The optical band gaps of LCC*x*, LSC*x*, and LSCM*x* compositions were determined by diffuse reflectance spectrophotometry analysis. Band gap energies were determined by applying the Kubelka–Munk method and the Tauc relation represented by eq 3.
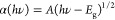
3

**Figure 6:**
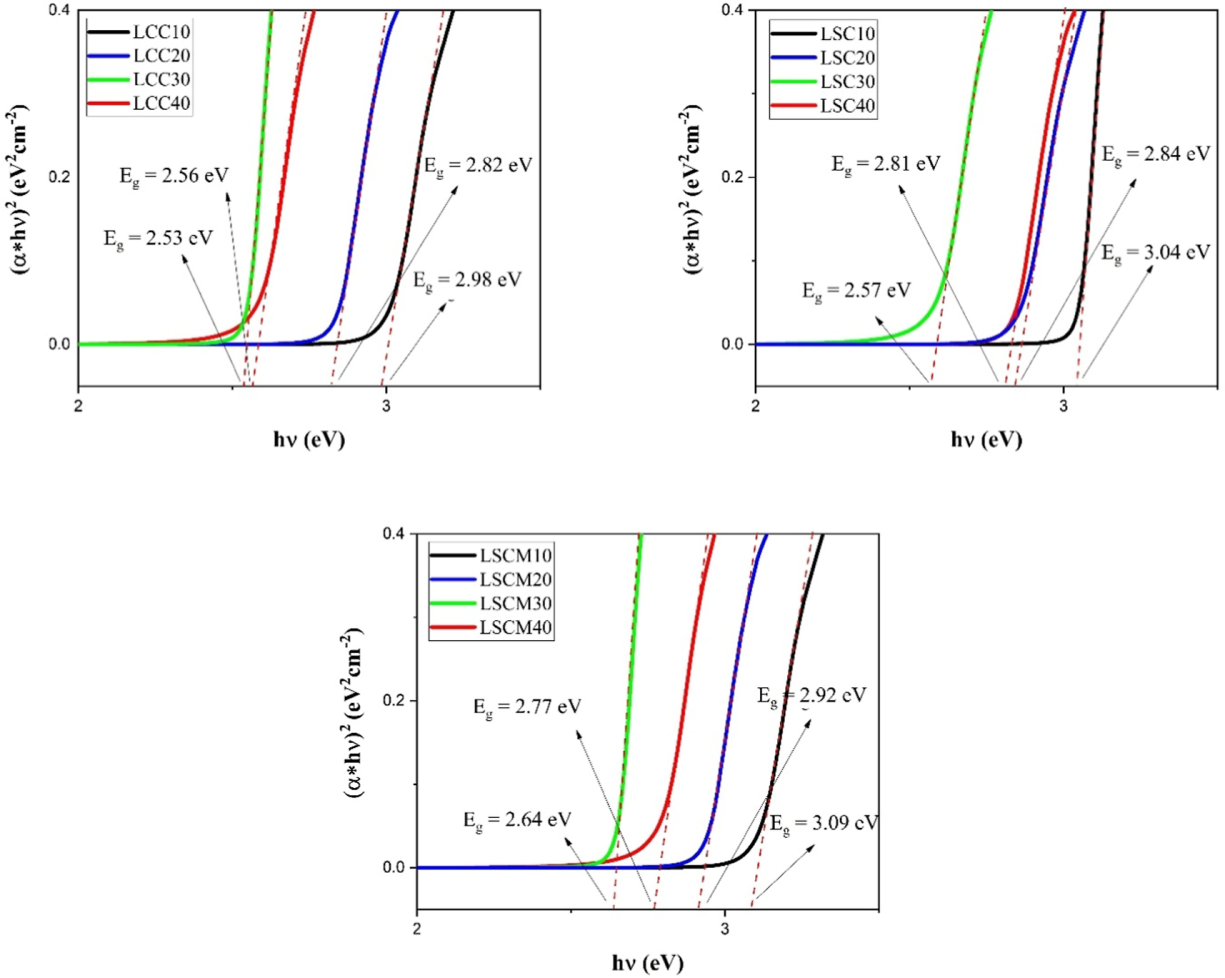
Tauc plots and optical band gap energies for LCC*x*, LSC*x*, and LSCM*x* measured by UV–vis reflectance spectrophotometry.

In this equation, α is the absorption coefficient, *h*ν is the incident photon energy, *A* is a constant, and *E*_g_ is the optical band gap of the material. *E*_g_ was extracted by plotting (α*h*ν)^1/*n*^ versus *h*ν and extrapolating the linear portion of the plot to (α*h*ν)^1/*n*^ = 0. In the above equation, *n* characterizes the type of transition process. For indirect transition processes, *n* = 2. Figure 6 shows the Tauc plots and optical band gap energies for LCC*x*, LSC*x*, and LSCM*x* measured by diffuse reflectance spectrophotometry analysis. It can be observed that for the LCC*x* compositions the optical band gap energy decreases proportionally as a function of calcium content for LCC10, LCC20, LLC30, and LCC40 with values of 3.12, 2.96, 2.84, and 2.81 eV, respectively. Similar optical band gap measurements were reported by Wang; the authors observed that the band gap energy decreased from 3.08 to 2.80 eV for LCC nanopowders with calcium substitution levels increasing from *x* = 0.1 to 0.4.^70^ A detailed explanation of how the new hole formation modifies the electronic band structure and band gap magnitude of these materials can be found in the DFT results (in the next section of this paper). In the same way, LSC*x* exhibited the same trend as that of the LCC*x* materials. With the change in the Sr content for LSC10, LSC20, LSC30, and LSC40, the gap energies of 3.07, 2.94, 2.86, and 2.84 eV, respectively, were measured (Figure 6). The band gap energy magnitude decreased as a function of the Sr concentration due to Cr^6+^ impurity absorption and the effects of Pauling electronegativity between Sr and oxygen. Finally, the same optical gap energy trends for the LSCM*x* materials were measured. For the LSCM10, LSCM20, LSCM30, and LSCM40 compositions, the band gaps were measured to be 3.10, 2.93, 2.85, and 2.82 eV, respectively. Sr^2+^ present in the A-site results in the formation of Cr^6+^; however, the divalent cation can induce the oxidation transition of Mn^3+^ to Mn^4+^ in the perovskite, which increases as a function of Mn concentration, leading in this way to the decrease in the band gap energy. No reports on LSC*x* and/or LSCM*x* optical band gap tuning studies were reported in the literature.

Table 5 summarizes the experimental and DFT optical band gap for LCC*x*, LSC*x*, and LSCM*x*. The discussion of the DFT modeling will be further discussed in the next section, but the optical gap data from this modeling are presented here for direct comparison to the experimental data. The effect of the Pauling electronegativity difference between metals and oxygen atoms in the ABO_3_ perovskite structure was proposed to predict band gap energy trends in relation with the A- or B-site elements. It was observed that the higher the difference in electronegativity between A- or B-site metal and oxygen, the higher the band gap of the perovskite material.^43^Table 6 shows the Pauling electronegativity difference of the substituent cations and oxygen in this work. The Pauling electronegativity difference between Ca–O (2.44) and Sr–O (2.49) is higher than that between La–O (2.34); in the same way, the difference between Mn–O (2.49) is higher than that between Cr–O (2.44). According to the Pauling difference approach, the optical band gap energies should increase as a function of Ca, Sr, and Mn concentrations; however, the direct measurements are not in agreement with this theory. This theoretical approach was observed for pure perovskites where the change on the A- or B-site can lead to the gap energy change. In this work, the gap energy change is mainly directed by the formation of hole formation; that is, the valence states generated by dopants in any A- or B-site significantly control the optical properties.

**Table 5:** Experimental and DFT Optical Band Gaps for LCC*x*, LSC*x*, and LSCM*x*

optical band gap (eV)		band gap (eV)	
composition	experimental	composition	DFT theoretical
LCC10	3.12	LaCrO_3_	2.91
LCC20	2.96	LCC12.5	2.83
LCC30	2.84	LCC25.0	2.77
LCC40	2.81	LCC37.5	2.47
LSC10	3.07	LSC12.5	2.80
LSC20	2.94	LSC25.0	2.70
LSC30	2.86	LSC37.5	2.69
LSC40	2.84	LSCM12.5	0.35
LSCM10	3.10	LSCM25.0	0.32
LSCM20	2.93	LSCM37.5	0.25
LSCM30	2.85		
LSCM40	2.82		

**Table 6:** Pauling Electronegativity Difference of the Substituent Cations and Oxygen

A-/B- cation	Pauling electronegativity	electronegativity difference with O
Cr	1.66	1.78
Mn	1.55	1.89
La	1.10	2.34
Ca	1.00	2.44
Sr	0.95	2.49

### DFT Band Structure Modeling

**Figure 7:**
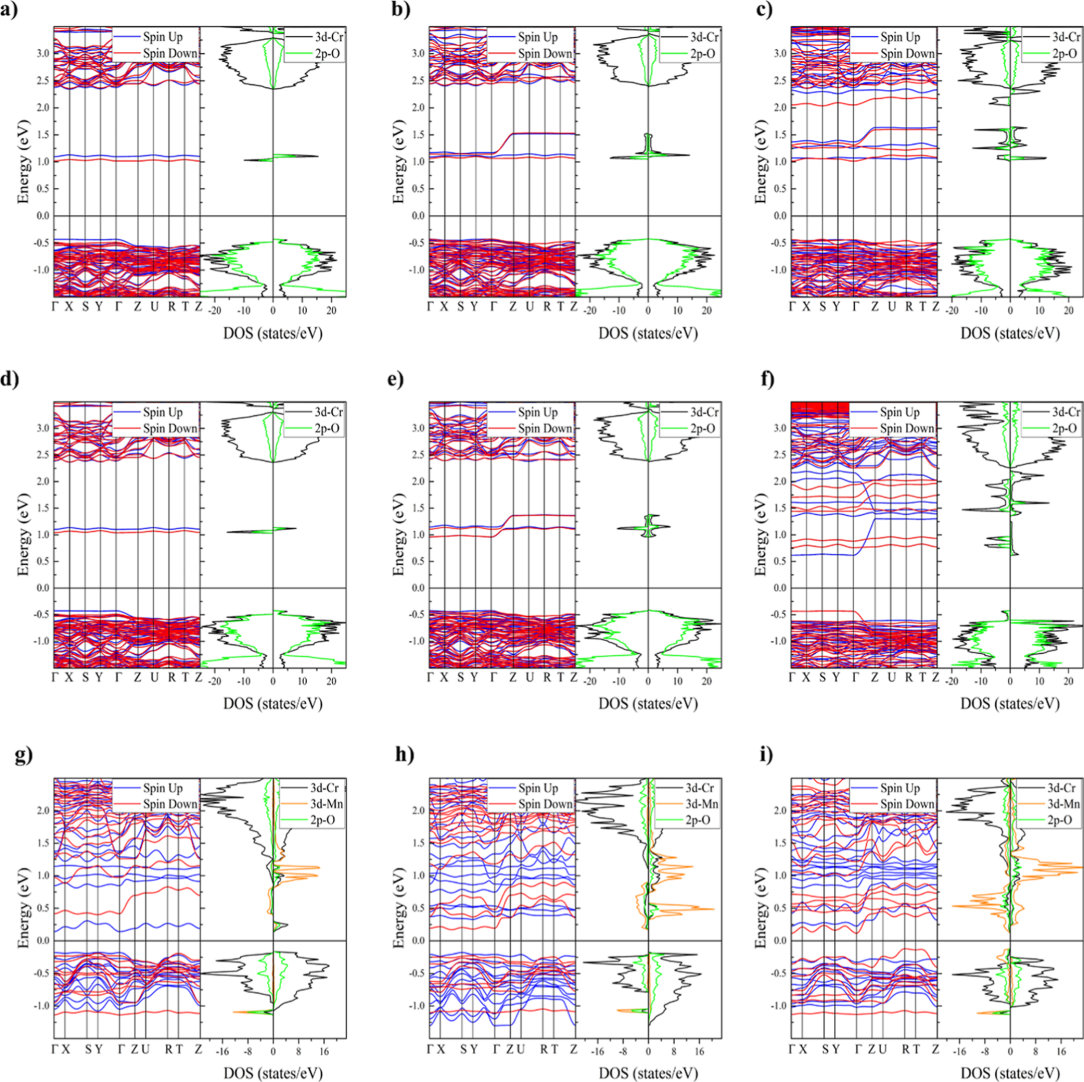
DFT calculated band structure and density of states for (a) LCC12.5; (b) LCC25.0; (c) LCC37.5; (d) LSC12.5; (e) LSC25.0; (f) LSC37.5; (g) LSCM12.5; (h) LSCM25.0; and (i) LSCM37.5.

Figure 7 shows the band structure and partial density of states (PDOS) calculated by the DFT model previously discussed for the 3d–Cr and 2p–O states on LCC*x* and LSC*x* and 3d–Cr, 2p–O, and 3d–Mn states on LSCM*x*. The theoretical *x* = 12.5, 25, and 37.5% substitution levels are slightly different than the experimental levels used in this work because only certain allowable atomic content is permitted for the supercell construction required. These states have a higher contribution near the Fermi level. With respect to the band structure and PDOS for LCC*x* and LSC*x*, it can be seen below the Fermi level in the minority and majority spin regions that the energy band peaks suggest a strong overlap between the 3d–Cr and the 2p–O orbitals. This indicates a strong hybridization between these two orbitals. On the other hand, unoccupied localized bands appear near and above the Femi level in the minority and majority spin regions, which are associated with a narrow polaron band; similar observations and conclusions were reported by ref (51). The location and shape of this defect state in the band gap are dependent on the dopant content; the number of defect bands increases with the dopant concentration due to the oxidation from Cr^3+^ to Cr^4+^ and Cr^6+^, as suggested in the experimental discussion. The peaks become less localized with a greater bandwidth because the electronic states are spatially more dispersed throughout the material. For LSC*x* at higher dopant concentrations, the defect band overlapped with the conduction band. It is important to note that as the dopant concentration was increased, the width of the band gap decreased, as observed in Table 5 and Figure 7, because the states are shifted to higher energy levels.

For the LSCM*x* compositions, for *x* = 12.5 and 25%, peaks of the energy bands in the minority and majority spin regions can be seen below and near the Fermi level, mainly caused by the 3d–Cr and 2p–O states, with a smaller contribution of the 3d–Mn states that are found in the lower part of the conduction band. However, for *x* = 37.5% below the Fermi level in the minority spin region, new states that emerge in 3d-Mn can be seen, which suggests that there could be a charge transfer from the Cr to the Mn across the O atoms when Mn is substituted in the structure. The content of Mn produced the broadening of this peak and generated the formation and increase in the hole concentration. There are many impurity levels that are generated near the Fermi level, and the band gap decreased to 0.25 eV, which could be due to the Cr^3+^ → Cr^4+^ → Cr^6+^ transitions. These theoretical results agree with our experimental observation and results reported by Yu,^49^ in Ca/Fe codoped LaCrO_3_. It is important to highlight that even if the DFT determined dopant concentration-band gap trend is the same as observed in the experimental results, discrepancies of the magnitudes of the band gap between the experimental and theoretical band gap values for samples LSCM12.5, LSCM25, and LSCM37.5 (Table 5) were obtained; these observations can be attributed to several factors. One of the main sources of discrepancy is the limitation of DFT in accurately representing the proportion of dopants within the crystal lattice, particularly at high substitution levels. In this study, the computational supercells were constructed under idealized periodic conditions, which do not fully capture local disorder, defect clustering, or nonstoichiometric effects present in real samples. Additionally, the potential influence of structural distortions induced by dopants, oxygen vacancies, and electron–phonon interactions—factors difficult to model within standard DFT frameworks—could also contribute to the deviation from experimental values. Moreover, localized electronic effects, such as the formation of small polarons and their coupling to defects, are often underestimated by conventional DFT functionals. Despite these discrepancies, the general trend of band gap narrowing with increased dopant concentration was well reproduced by the theoretical calculations, validating the overall predictive capability of the model.

**Figure 8:**
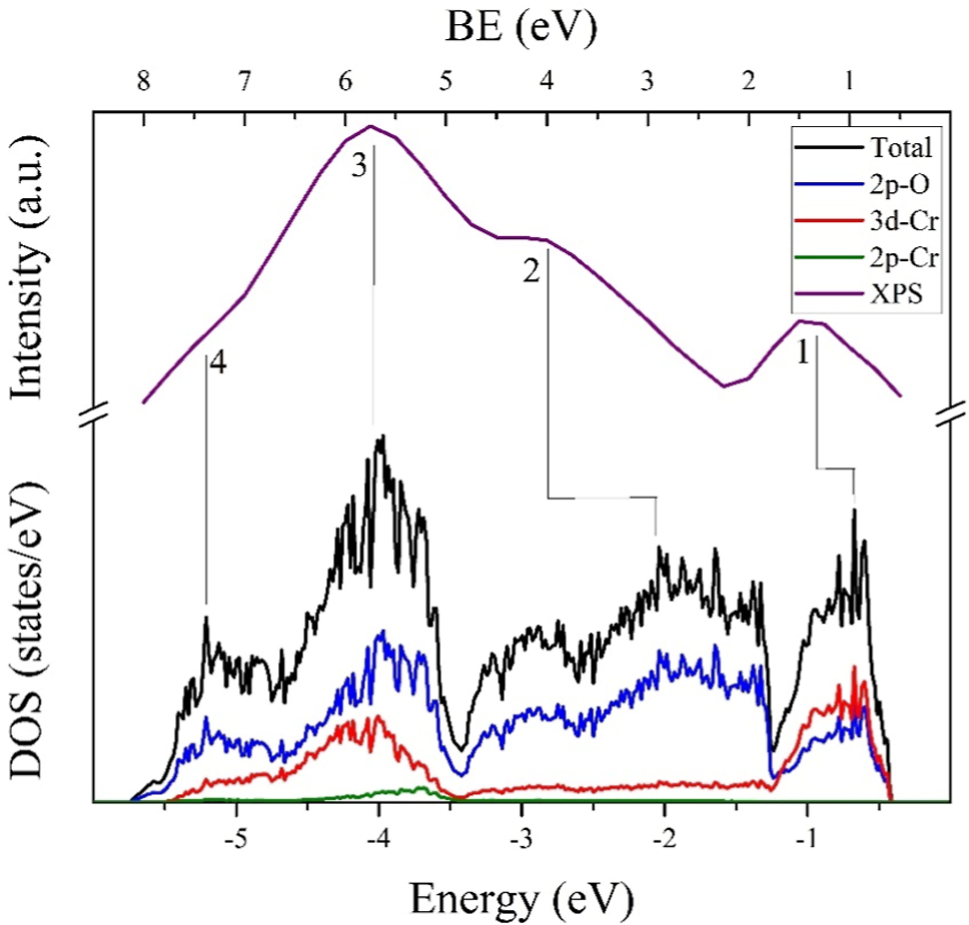
Comparison of the total and PDOS of the 3d–Cr, 2p–Cr, and 2p–O states of LSC*x* (*x* = 25%) and experimental XPS spectra for LSC*x* (*x* = 20%).

Figure 8 shows the comparison of the total and PDOS of the 3d–Cr, 2p–Cr, and 2p–O states of LSC*x* (*x* = 25%) and experimental XPS spectra. In order to align the peaks of the theoretical density of states with those of the experimental XPS, the energy scale of the density of states was reduced. It is worth noting that despite the concentration of Sr in the theoretical calculations being higher (25%) than that used in the experimental measurements (20%), our theoretical results agree with the experimental XPS spectra. This guarantees that the computational methods and DFT functional employed correctly reproduce the properties of the compounds under study. Taking the above into account, it can be observed that the peaks of the total density of states and XPS spectra are mainly formed by electrons from the 3d–Cr and 2p–O states, with a lesser contribution from the 2p–Cr states.

Complementary experimental evidence of p-type positive hole formation, supporting the activation of the polaron hopping conductivity mechanism, was obtained through DC electrical conductivity measurements using the four-point method as a function of temperature and dopant concentration (provided in the Supporting Information section). The formation of localized holes near the dopant sites, resulting from the oxidation of Cr^3+^ to Cr^4+^ and Cr^6+^, occurs both at the surface and within the bulk of the material due to diffusion-driven processes at high temperatures. These holes enhance the small polaron hopping mechanism, thereby facilitating charge transport throughout the material; this indicates that increasing the concentration of substituents cations in the A- or the B-site of LaCrO_3_, the conductivity increases proportionally. These electrical conductivity behavior and trends are consistent with the narrowing of the band gap observed in both experimental and DFT results, underscoring the critical role of defect states—created by oxygen vacancies and dopants—in tuning the electronic structure and electrical properties of LaCrO_3_-based materials.

## Conclusions

This study included a systematic study of the valence state transitions and optical band gap of lanthanum chromite compositions with multivalent substitutions. Unlike many previous works, a singular processing method was consistently used to fabricate all compositions, where the Pechini-like sol–gel method yielded high phase purity and density ceramics (>95% theoretical density). The transition states were characterized using XPS. XPS revealed that increased concentrations of divalent dopants raised Cr^4+^ levels under oxidizing conditions while reducing atmospheres promoted the transition from Cr^4+^ to Cr^3+^ due to the formation of oxygen vacancies. While this behavior had been previously assumed, this study provides the first direct evidence of these transitions using XPS as a function of atmosphere, offering quantifiable insights into surface valence state changes. These findings highlight the significant influence of atmospheric conditions on the valence state transitions, specifically demonstrating how different dopant levels and environmental conditions alter the ratio of Cr^3+^/Cr^4+^. This change impacts the polaron hopping mechanism, thereby affecting the overall electronic conduction behavior in lanthanum chromite. The optical band gap for these same compositions was measured by using UV–vis diffuse reflectance spectroscopy. The measurements showed that increasing divalent or trivalent substitution in LaCrO_3_ led to a decrease in band gap energy. This decrease was attributed to the Cr^4+^ and Cr^6+^ impurity absorption or the formation of positive hole bands. These results mark the first experimental documentation of such trends in these systems. DFT calculations further complemented the experimental findings by modeling the band structures and density of states for Ca-, Sr-, and Sr/Mn-doped LaCrO_3_. The theoretical calculations showed consistent trends with the experimental optical band gap measurements, revealing that higher dopant concentrations correlate with a reduced band gap. The DFT results indicated that the valence and conduction bands are primarily populated by Cr and O states. Additionally, the introduction of dopants created new defect states above the valence band, which contributes to the observed reduction in the band gap magnitude. This comprehensive study provided specific insights into the effects of multivalent doping on the optical and electronic properties of lanthanum chromite perovskites. For the first time, Cr^4+^ to Cr^3+^ transitions were experimentally documented in Sr^2+^/Mn^3+^ codoped samples, and it was quantified how divalent and trivalent dopants directly influence both valence states and the optical band gap. These results significantly advance the understanding of dopant-induced modifications in the band structure and their implications for tuning the material’s electronic properties for potential applications.

## Supporting Information Available

XRD, shift in diffraction peak, and temperature-dependent electrical conductivity (PDF)
