# Supplementary material for: Doping Effects on Multivalence States, Electronic Structure, and Optical Band Gap in LaCrO3 under Varied Atmospheres: An Integrated Experimental and Density Functional Theory Study
Source: ACS Appl Electron Mater. 2025 Mar 7;7(6):2515–28. doi: 10.1021/acsaelm.4c02359 (PMC11948326; doi:10.1021/acsaelm.4c02359)
Supplement: Supplementary file 1 — el4c02359_si_001.pdf [file el4c02359_si_001.pdf]

SUPPORTING INFORMATION

Doping Effects on Multivalence States, Electronic Structure, and Optical Band Gap in $LaCrO_3$ under Varied Atmospheres: An Integrated Experimental and DFT Study

Edward M. Sabolsky*[1], Javier A. Mena[1], Víctor Mendoza-Estrada[2,3,4], Rafael González-Hernández[2], Katarzyna Sabolsky[1], Konstantinos Sierros[1]

[1]Department of Mechanical and Aerospace Engineering, West Virginia University, Morgantown, WV 26506, USA

[2]Grupo de Investigación en Física Aplicada, Departamento de Física, Universidad del Norte, Barranquilla, Colombia

[3]Institución Universitaria de Barranquilla, Facultad de Ciencias, Educación, Artes y Humanidades, Barranquilla, Colombia

[4]Colegio San José, Área de Ciencias Naturales, Puerto Colombia, Colombia

*Corresponding author: Ed.Sabolsky@mail.wvu.edu

High-density ceramic pellets were achieved through a reactive sintering process as outlined in the experimental section. XRD analysis was performed on the strontium-doped lanthanum chromite (LSCx) compositions with substitution levels ranging from 10% to 40%, as shown in **Fig. S1**. The XRD diffractograms indicate that all compositions (LSC10, LSC20, LSC30, and LSC40) exhibit diffraction peaks corresponding to the lanthanum chromite orthorhombic perovskite structure with space group Pnma [1] (JCPDS card #24-1016). The prominent diffraction planes observed include (110), (112), (022), (004), (114), (024), (224), (143), and (332), confirming the formation of a single phase without detectable secondary phases or unreacted oxides. The absence of impurity peaks is clear evidence of successful incorporation of Sr^{2+} into the A-site of the $LaCrO_3$ perovskite structure, maintaining structural integrity across all substitution levels.

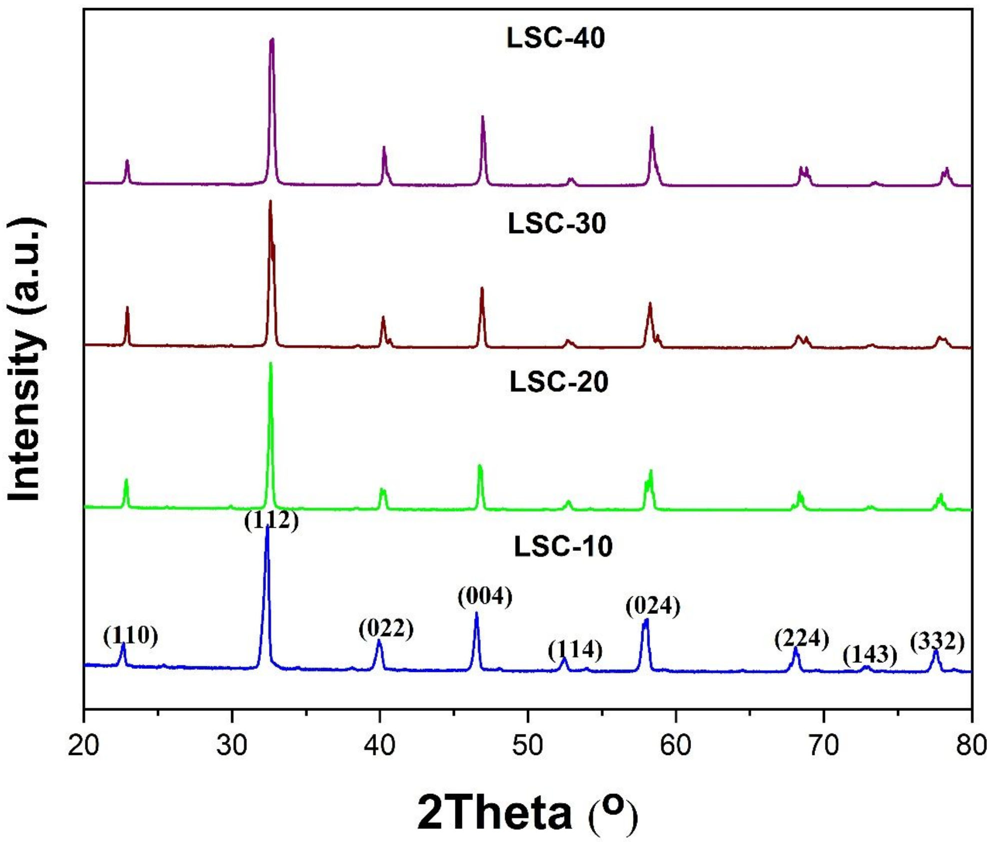

Fig. S1 XRD patterns of Sr-doped $LaCrO_3$ (LSCx) compositions with varying Sr concentrations (10% to 40%) after sintering at 1600 °C.

Fig. S2 highlights the systematic rightward shift of the (110) diffraction peak as the strontium concentration increases. This shift indicates a gradual contraction of the unit cell, which, at first glance, may seem contradictory given that Sr^{2+} (ionic radius 1.44 Å [2]) is larger than La^{3+} (1.36 Å [3]). The unexpected contraction can be attributed to simultaneous oxidation occurring at the B-site, where Cr^{3+} is partially oxidized to Cr^{+4} and Cr^{+6}. These higher oxidation states (with smaller ionic radii: Cr^{+4} = 0.50 Å, Cr^{+6} = 0.44 Å) counteract the A-site expansion and lead to the observed net decrease in lattice parameters. This phenomenon of cell contraction due to simultaneous A-site and B-site effects has been reported in similar perovskite systems and is an indicator of the

successful charge compensation mechanism induced by Sr^{+2} doping. Same pattern trends were observed for LCCx compositions (divalent cation substitution in the lanthanum A-site).

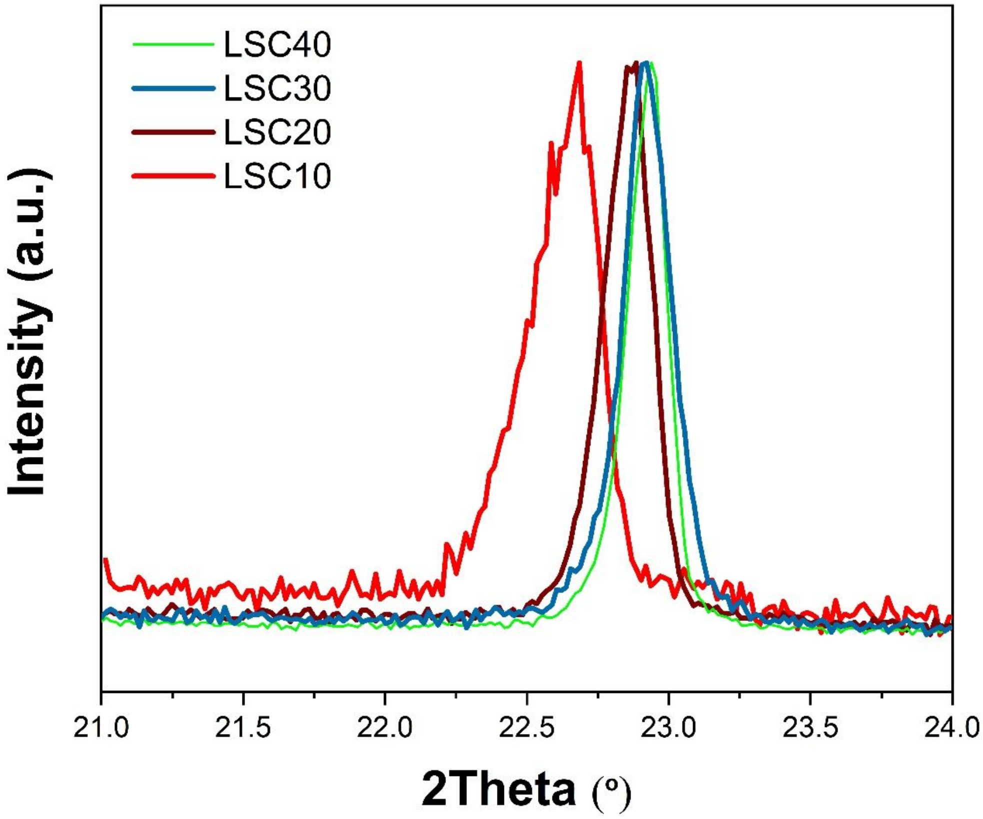

Fig. S2 Shift in the (110) diffraction peak of LSCx compositions as a function of Sr dopant concentration, indicating unit cell contraction.

As shown in **Fig. S3**, the XRD diffractograms for LSCM10, LSCM20, LSCM30, and LSCM40 reveal diffraction patterns corresponding to the same orthorhombic perovskite structure (*Pnma*, JCPDS card #24-1016) observed in LSCx compositions. Up to 30% Mn substitution, all compositions remain single-phase, confirming the formation of a stable solid solution. However, at 40% Mn substitution (LSCM40), peak splitting becomes evident in several crystallographic

planes, including (022), (004), and (114). This splitting suggests the emergence of a secondary perovskite phase, possibly associated with phase segregation due to the high dopant concentration [4]. The reduction in lattice parameters as the Mn content increases correlates with the oxidation processes occurring at the B-site, specifically $Cr^{+3} \rightarrow Cr^{+4} \rightarrow Cr^{+6}$ and $Mn^{+3} \rightarrow Mn^{+4}$. The smaller ionic radii of Mn^{+4} (0.53 Å) and Cr^{+4} (0.50 Å) relative to their lower oxidation states contribute to the observed structural contraction, despite the larger ionic radius of Sr^{+2}.

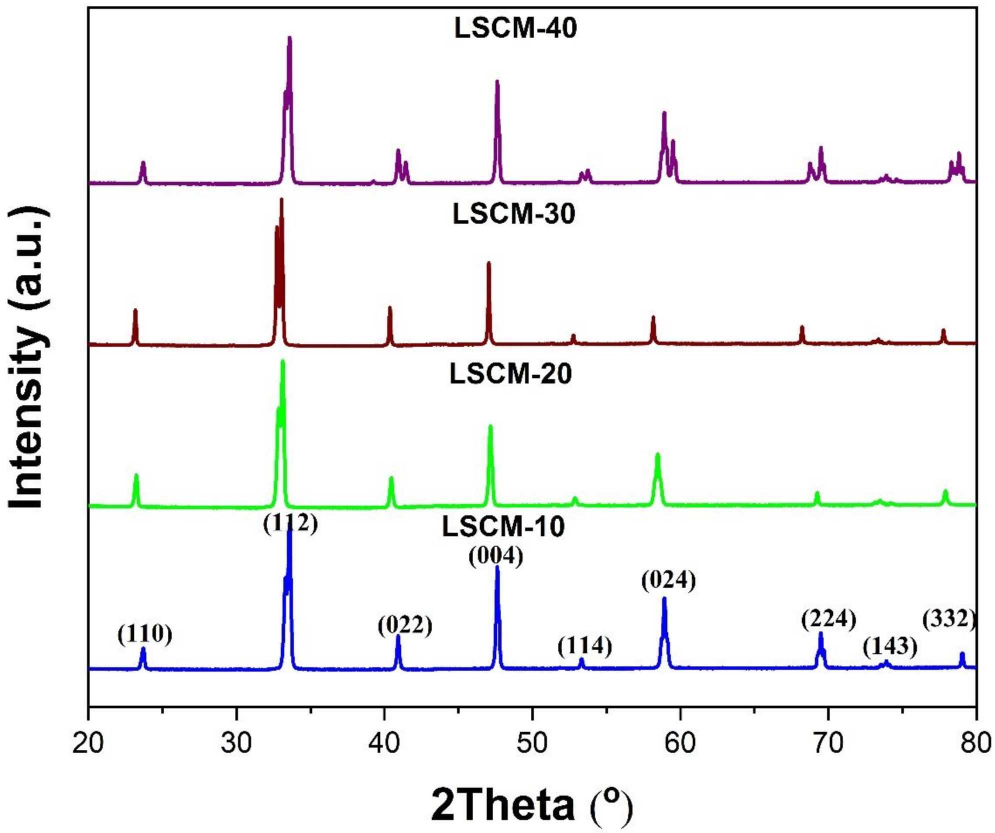

Fig. S3 XRD patterns of Sr/Mn co-doped $LaCrO_3$ (LSCMx) compositions with varying Mn concentrations (10% to 40%) after sintering at 1600 °C.

The DC conductivity measurements for Ca-doped $LaCrO_3$ (LCCx) compositions are presented in Fig. S4, showing a clear and consistent increase in electrical conductivity as both the dopant concentration and temperature rise. The conductivity at high temperatures (>1000 °C) is significantly higher for LCC40 compared to LCC10, highlighting the direct influence of Ca substitution on the transport properties of the material. The enhanced conductivity is attributed to the formation of p-type positive holes due to the oxidation of Cr^{+3} to Cr^{+4}. The XPS measurements, as previously discussed, confirm the coexistence of these oxidation states, supporting the hypothesis that Ca^{+2} substitution triggers a charge compensation mechanism involving the generation of Cr^{+4}. These oxidized states introduce localized holes, which facilitate charge transport through the small polaron hopping mechanism. The narrowing of the optical bandgap, observed through both experimental UV-Vis spectroscopy and DFT calculations, further supports this mechanism. As the bandgap decreases with increased dopant concentration, the energy barrier for charge hopping between Cr ions is reduced, enhancing the overall conductivity. The strong correlation between bandgap narrowing, mixed valence states (evidenced by XPS), and conductivity trends highlights the role of defect engineering in tailoring the electronic properties of LCCx materials, especially at elevated temperatures.

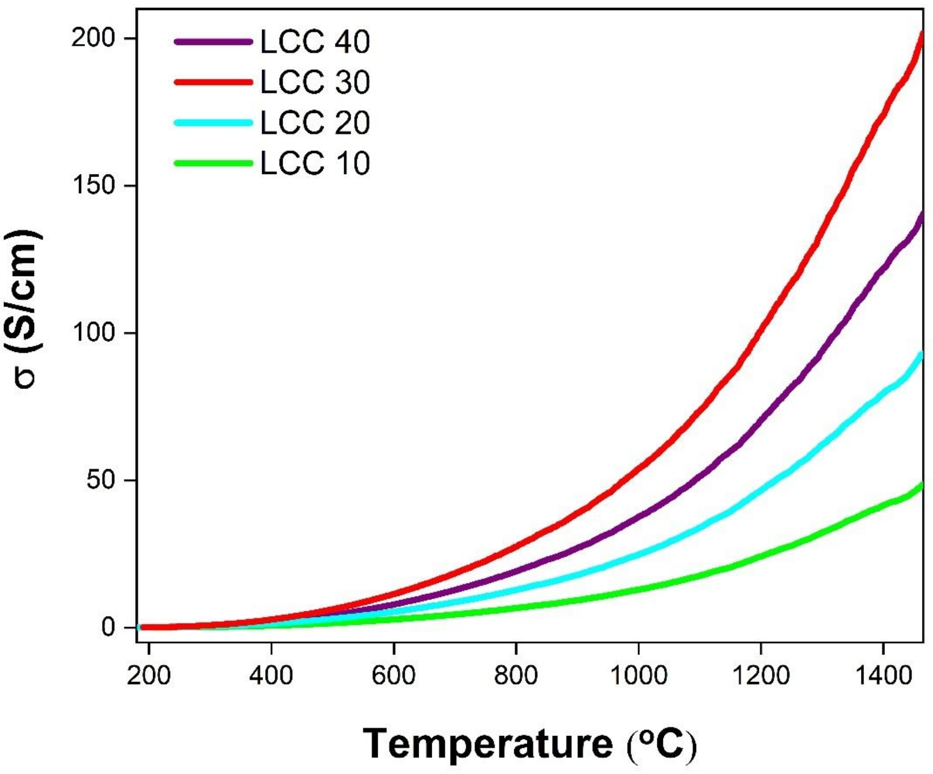

Fig. S4 Temperature-dependent electrical conductivity (σ) of Ca-doped $LaCrO_3$ (LCCx) compositions measured via the DC four-point method.

Fig. S5 illustrates the DC conductivity trends for Sr/Mn co-doped $LaCrO_3$ (LSCMx) compositions. Similar to LCCx, the conductivity increases with both temperature and dopant concentration, with LSCM40 showing the highest conductivity values. However, compared to the LCCx series, the overall conductivity of LSCMx compositions is lower, reflecting the combined effects of Sr and Mn substitution on the charge transport mechanism. The incorporation of Sr^{+2} and Mn^{+3} leads to a complex charge compensation mechanism involving the oxidation of Cr^{+3} to Cr^{+4} and Mn^{+3} to Mn^{+4}, as confirmed by XPS spectra. The localized holes generated near both Cr and Mn sites contribute to the small polaron hopping mechanism, although the competing effects of Sr and Mn

substitutions on the lattice strain and defect density partially limit the overall conductivity compared to the LCCx series. Furthermore, the narrowing of the bandgap in LSCMx compositions, observed through experimental measurements and DFT predictions, aligns with the conductivity trends. The reduction in bandgap facilitates easier charge transfer, but the formation of secondary phases at higher Mn concentrations (as evidenced by XRD peak splitting in LSCM40) may introduce scattering centers that slightly hinder the charge transport efficiency.

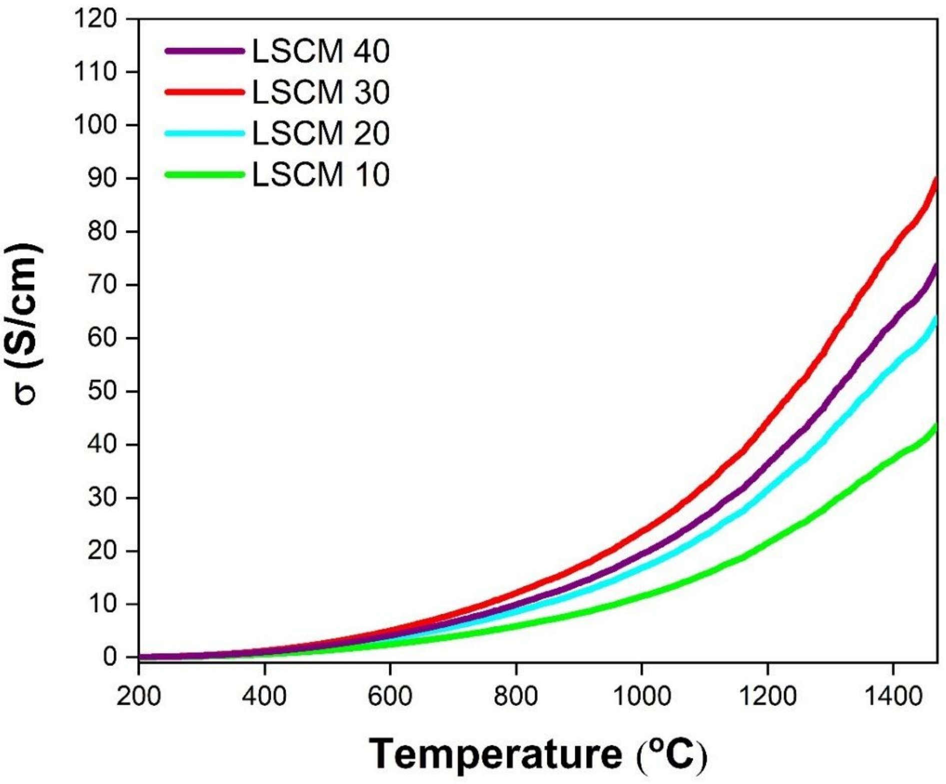

Fig. S5 Temperature-dependent electrical conductivity (σ) of Sr/Mn co-doped $LaCrO_3$ (LSCMx) compositions measured via the DC four-point method.

The electrical conductivity results are strongly correlated with the structural and electronic modifications induced by dopant incorporation. The observed trends can be explained by the

interplay between mixed valence states, bandgap narrowing, and defect formation within the crystal lattice. XPS analysis provides clear evidence of the coexistence of Cr^{3+}, Cr^{4+} oxidation states, which are directly responsible for the formation of p-type positive holes. These localized holes act as charge carriers, activating the small polaron hopping mechanism and enhancing electrical conductivity, particularly at high temperatures. The narrowing of the optical bandgap, as revealed by both experimental UV-Vis measurements and DFT calculations, further facilitates this charge transport mechanism. As the bandgap decreases with increasing dopant concentration, the energy barrier for hole hopping is reduced, allowing for more efficient charge transfer between Cr ions. This reduction in bandgap is not only a result of structural changes but also a reflection of the electronic states introduced by oxygen vacancies and dopant-related defects. Defect formation and structural modifications also play a crucial role in determining the overall transport properties. XRD analysis shows that dopant incorporation leads to unit cell contraction, likely driven by the oxidation of Cr^{+3} and Mn^{+3} to their higher oxidation states with smaller ionic radii. This contraction affects the defect density within the lattice and influences the availability of charge carriers. In the case of LSCMx compositions, secondary phase formation at higher Mn concentrations introduces scattering centers, slightly limiting charge transport compared to the LCCx series. Together, the mixed valence states, bandgap narrowing, and defect engineering provide a comprehensive framework for understanding the enhanced electrical conductivity in $LaCrO_3$-based perovskites. The synergy between these factors demonstrates the effectiveness of controlled doping in tuning the electronic structure and optimizing the material's performance for high-temperature applications such as solid oxide fuel cells and thermoelectric devices.

REFERENCES

[1] Liu, X.; Su, W.; Lu, Z.; Liu, J.; Pei, L.; Liu, W.; He, L. Mixed Valence State and Electrical Conductivity of $La_{1-x}Sr_xCrO_3$. *J. Alloys Compd.* **2000**, *305*, 21–23. https://doi.org/10.1016/S0925-8388(00)00735-0.

[2] Shannon, R. D. Revised Effective Ionic Radii and Systematic Studies of Interatomic Distances in Halides and Chalcogenides. *Acta Crystallogr., Sect. A* 1976, *32*, 751–767. https://doi.org/10.1107/S0567739476001551.

[3] Choi, B. H.; Park, S.; Park, B. K.; Chun, H. H.; Kim, Y. Controlled Synthesis of $La_{1-x}Sr_xCrO_3$ Nanoparticles by Hydrothermal Method with Nonionic Surfactant and Their ORR Activity in Alkaline Medium. *Mater. Res. Bull.* 2013, *48*, 3651–3656. https://doi.org/10.1016/j.materresbull.2013.04.084.

[4] Song, Y.; Zhong, Q.; Tan, W. Synthesis and Electrochemical Behavior of Ceria-Substituted LSCM as a Possible Symmetric Solid Oxide Fuel Cell Electrode Material Exposed to H_2 Fuel Containing H_2S. *Int. J. Hydrogen Energy* 2014, *39*, 13694–13700. https://doi.org/10.1016/j.ijhydene.2014.03.179.
